# The Alteration of Lipid Metabolism in Burkitt Lymphoma Identifies a Novel Marker: Adipophilin

**DOI:** 10.1371/journal.pone.0044315

**Published:** 2012-08-31

**Authors:** Maria R. Ambrosio, Pier P. Piccaluga, Maurilio Ponzoni, Bruno J. Rocca, Valeria Malagnino, Monica Onorati, Giulia De Falco, Valeria Calbi, Martin Ogwang, Kikkeri N. Naresh, Stefano A. Pileri, Claudio Doglioni, Lorenzo Leoncini, Stefano Lazzi

**Affiliations:** 1 Department of Human Pathology and Oncology, Anatomical Pathology Section, University of Siena, Siena, Italy; 2 Molecular Pathology Laboratory, Haematopathology Unit, Department of Haematology and Oncology "L. and A. Seràgnoli", S. Orsola-Malpighi Hospital, University of Bologna, Bologna, Italy; 3 Pathology Unit, Department of Oncology, University Scientific Institute San Raffaele, Milan, Italy; 4 Saint Mary Hospital, Lacor Gulu, Uganda; 5 Department of Histopathology, Hammersmith Hospital Campus, Imperial College, London, United Kingdom; University of Massachusetts Medical School, United States of America

## Abstract

**Background:**

Recent evidence suggests that lipid pathway is altered in many human tumours. In Burkitt lymphoma this is reflected by the presence of lipid droplets which are visible in the cytoplasm of neoplastic cells in cytological preparations. These vacuoles are not identifiable in biopsy section as lipids are “lost” during tissue processing.

**Methods and Results:**

In this study we investigated the expression of genes involved in lipid metabolism, at both RNA and protein level in Burkitt lymphoma and in other B-cell aggressive lymphoma cases. Gene expression profile indicated a significant over-expression of the adipophilin gene and marked up-regulation of other genes involved in lipid metabolism in Burkitt lymphoma. These findings were confirmed by immunohistochemistry on a series od additional histological samples: 45 out of 47 BL cases showed strong adipophilin expression, while only 3 cases of the 33 of the not-Burkitt lymphoma category showed weak adipophilin expression (p<0.05).

**Conclusions:**

Our preliminary results suggest that lipid metabolism is altered in BL, and this leads to the accumulation of lipid vacuoles. These vacuoles may be specifically recognized by a monoclonal antibody against adipophilin, which may therefore be a useful marker for Burkitt lymphoma because of its peculiar expression pattern. Moreover this peptide might represent an interesting candidate for interventional strategies.

## Introduction

Burkitt lymphoma (BL) is listed in the World Health Organization (WHO) classification of tumours of haematopoietic and lymphoid tissues as an ‘aggressive B-cell non-Hodgkin lymphoma’(B-NHL) [1]. Based on epidemiological features, BL is subdivided into three variants: endemic, sporadic and immunodeficiency-associated. While the Epstein–Barr virus (EBV) is associated with 98% of the endemic BL, it is seen only in 20% of sporadic cases, and 30–40% of immunodeficiency-associated cases [2]. Histologically BL is characterized by a monotonous infiltrate of medium-sized blastic lymphoid cells that show round nuclei with clumped chromatin and multiple nucleoli and by the presence of a ‘starry sky’ pattern. On fine needle aspiration cytology (FNAC), BL shows very typical lipidic vacuoles in the cytoplasm of lymphoid cells which represents a diagnostic hallmark [3]. Unfortunately, these vacuoles cannot be seen on histological preparations because during waxing and fixation they are largely lost from tissues, and a combination of several diagnostic techniques (such as morphology, immunophenotyping or genetic analysis) is necessary to achieve the diagnosis of BL [1]. The tumour cells are positive for CD79a, CD20, CD10, BCL6, CD38, and are negative for BCL2, Mum-1, CD44 and CD138. The proliferation fraction measured by Ki-67 is nearly 100% [2]. At molecular level, BL is characterized by the chromosomal rearrangement of *MYC*, in the form of reciprocal translocation juxtaposing the *MYC* gene at 8q24, to the immunoglobulin heavy chain (*IGH*) locus at 14q32, or the *IGK* (2p11) or *IGL* (22q11) light chain loci [2].

The presence of lipid vacuoles in the cytoplasm of BL cells on FNAC may suggest that biosynthesis of lipids and other macromolecules is altered in BL. Previous study has found that lipogenic pathway is activated in some tumours (i.e. hepatocellular carcinoma, clear cell renal carcinoma, adenocarcinoma of the colon, sebaceous tumours) [4]–[5]. In contrast to normal cells, neoplastic cells rely mainly on anaerobic glycolysis, a phenomenon known as Warburg effect [5]. To sustain the rapid proliferation and to counteract the hostile environment, cells must increase the rate of metabolic reactions to provide adenosine triphosphate, lipids, nucleotides and amino acids necessary for daughter cell production [6]–[7]. A recent paper on lipid metabolism in B-NHL has shown dysregulation of fatty acid synthesis and increase of glycolysis in these tumours, suggesting fatty acid synthase enzyme as a candidate for molecular target therapy [8].

**Figure 1:**
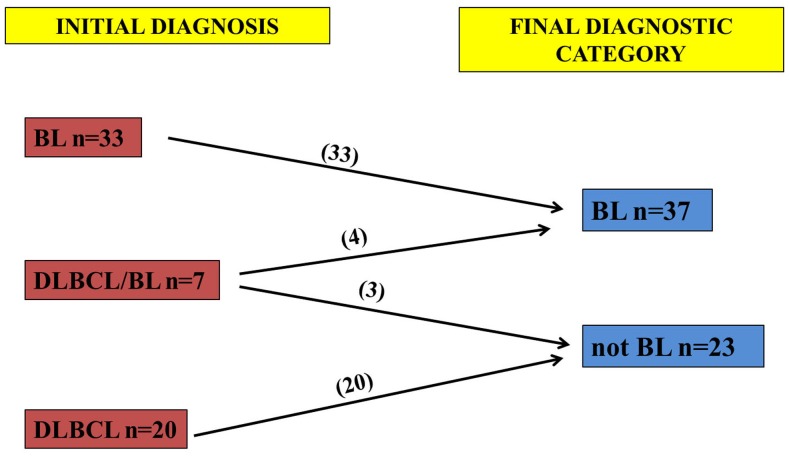
Classification of cases according to the algorithm proposed by Naresh *et al*. After the revision of 43 BL, 30 DLBCL and 7 DLBCL/BL cases, 47 BL and 33 not-BL cases were identified.

The present study was designed to analyse the lipid metabolism in BL and to identify a novel possible marker. We investigated by gene expression profile (GEP) the genes involved in the lipogenic pathway in 13 BL and 20 diffuse large B-cell lymphoma (DLBCL) cases. We observed differences in lipid metabolism between BL and DLBCL and identified adipophilin (adipocyte-differentiation-related protein) as the only member of the Perilipin, Adipholin, Tail-interacting protein of 47 kDa (TIP47) (PAT)-proteins family strongly expressed in BL [9]. The GEP results were validated on a series of additional cases classified according to the recent algorithm proposed by Naresh *et al*. [10] by immunohistochemistry, using a monoclonal antibody against the adipophilin to confirm lipid accumulation in standard formalin-fixed paraffin-embedded samples [4]. We observed that 45 out of 47 cases of BL showed positivity for adipophilin whereas only 3 out of 33 cases of the not-BL category were adipophilin-positive. These results suggest adipophilin as a novel marker for BL.

**Table 1:** Genes involved in lipid metabolism in BL other than PLIN2.

Probe Set ID	p-value	Unigene (Avadis)	Gene Symbol	Gene Title	Regulation	Entrez Gene
200831_s_at	0,006725255	**Hs.558396**	SCD	stearoyl-CoA desaturase(delta-9-desaturase)	down	6319
211708_s_at	9,94E-04	**Hs.558396**	SCD	stearoyl-CoA desaturase(delta-9-desaturase)	down	6319
212218_s_at	0,033671293	**Hs.83190**	FASN	fatty acid synthase	up	2194
220232_at	0,009198004	**Hs.379191**	SCD5	stearoyl-CoA desaturase 5	up	79966
223437_at	3,85E-06	**Hs.103110**	PPARA	peroxisome proliferator-activatedreceptor alpha	down	5465
223438_s_at	1,79E-05	**Hs.103110**	PPARA	peroxisome proliferator-activatedreceptor alpha	down	5465
226978_at	3,51E-07	**Hs.103110**	PPARA	peroxisome proliferator-activatedreceptor alpha	down	5465
231768_at	0,001676206	**Hs.414880**	USF1	upstream transcription factor 1	up	7391

## Materials and Methods

### Ethics Statement

Ethics approval for this study was obtained from the Institutional Review Board at the University of Siena (Italy) and from the Ethics and Research Committee at the Lacor Hospital (Uganda). Informed written consent was obtained in all cases.

### Case Selection

A total of sixty formalin-fixed and paraffin-embedded specimens were investigated at the Department of Human Pathology and Oncology, Anatomical Pathologic Section, University of Siena, Italy; the cases had been accrued locally and from Lacor Hospital, Uganda. The initial diagnosis of these cases was: BL in 43, diffuse large B-cell lymphoma (DLBCL) in 30 and B-cell lymphoma unclassifiable with features intermediate between DLBCL and BL (DLBCL/BL) in 7 [11].

**Figure 2:**
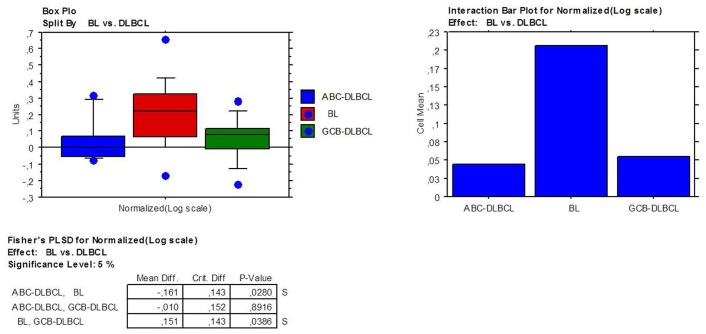
ADPF gene over-expression in BL compared to DLBCL. Box plots represent the values distribution across the three considered categories (A); the extremities of the boxes indicate the first and third quartile. The horizontal lines inside the boxes indicate median values; outlier values are indicated by blue dots. Histograms represent the mean expression values in the three groups (B).

**Figure 3:**
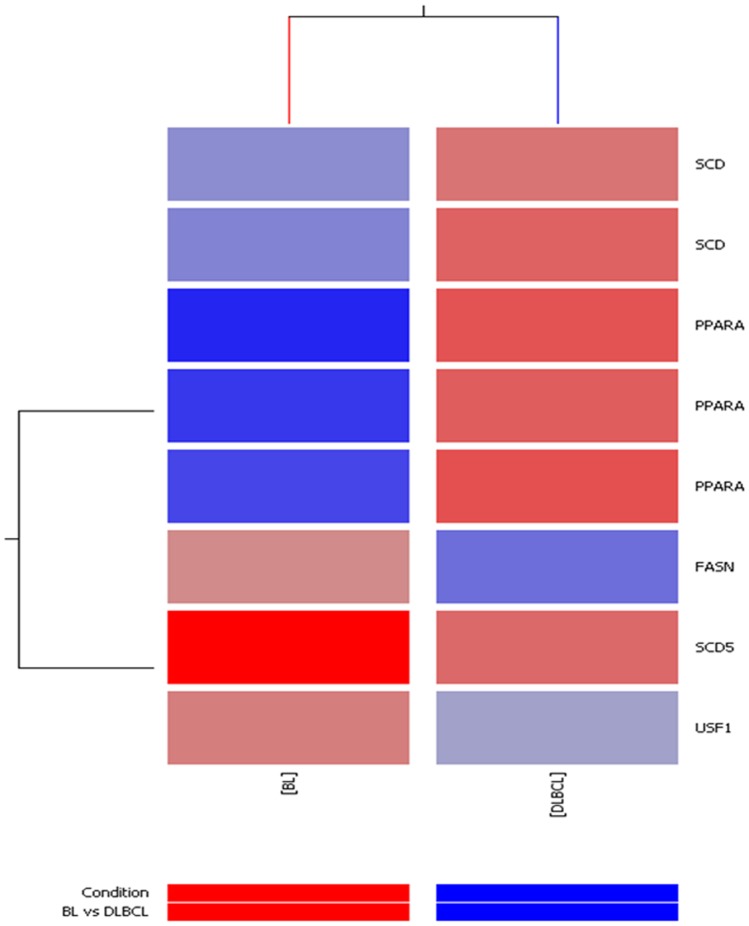
Over-expression of genes involved in lipid metabolism in BL. *SCD5*, *FASN* and *USF1* are up-regulated in BL; *SCD* and *PPARA* are up-regulated in DLBCLs.

### Histological and Immunohistochemical Studies

The slides were reviewed by two expert haematopathologists (LL, SL) and classified according to the scoring system recently designed by Naresh *et al.* [10] for aggressive B-cell lymphomas which distinguishes BL and not-BL. In fact, from a practical standpoint it is more beneficial to focus on two categories, given that the prognosis for the DLBCL/BL is uniformly poor [10]. According to the algorithm, we identified 47 BL and 33 not-BL cases (Figure 1). Histological sections (4-µm thick) were placed on positively charged glass slides (ProbeOn Plus; Fisher Scientific, Pittsburgh, PA, USA). The staining was performed on Bond Max automated immunostainer (Leica Microsystem, Bannockburn, IL, USA), with adipophilin antibody (pre-dilute AP 125; ProgenBiotechnik GmbH Maabstrasse Heidelberg, Germany) with controls in parallel. No epitope retrieval was used. Ultravision Detection System using anti-Polyvalent HRP (LabVision, Fremont, CA, USA) and diaminobenzidine (DAB, Dako, Milan-Italy) as chromogen was used. The pattern of immunostaining as well as the labelling intensity was evaluated independently in each of the 80 cases. The statistical association between the distribution of expression of adipophilin and the diagnostic category (BL and not-BL) was analysed using χ^2^–test and Fisher’s exact test, with *P*<0.05 considered as being statistically significant.

**Table 2:** Adipophilin expression, including intensity of staining and pattern of expression.

Histotype	N. positive (%)	N. negative (%)	Intensity of staining	Pattern of expression
**BL**	45 (96%)	2 (4%)	strong	single or multiple droplets into the cytoplasm, clusteringaround the outer nuclear membrane
**not-BL**	3 (9%)	30 (91%)	weak	single, very small droplets into the cytoplasm

BL: Burkitt lymphoma; N.: number of cases; %: percentage.

### Gene Expression Analysis

We analyzed GEP data of 13 BL and 20 DLBCL (10 GCB-type and 10 ABC-type), previously generated by using the Affymetrix HG-U133 2.0 plus microarray (Affymetrix, Inc. http://www.affymetrix.com/support/index.affx) and available at http://www.ncbi.nlm.nih.gov/geo/query/acc.cgi?acc=GSE26673 (GSE26673). For technical details, see reference 12 [12]. In particular, we focused on the expression of *ADFP (PLIN2)*, identified by a specific probe set in the HG-U133 2.0 plus GeneChip (209122_at). In addition, we studied the expression of genes whose activity is known to be related to lipid metabolism, such as, *SCD, SCD5, FASN, USF1, PPARA,* and represented in the microarray by the following probe sets: 200831_s_at, 211708_s_at, 220232_at, 212218_s_at, 231768_at, 223437_at, 223438_s_at, 226978_at. Further details on patients as well as on GEP generation were previously reported [9]. Supervised analysis and hierarchical clustering were performed as previously described by using GeneSpring GX11.0 platform (Agilent, USA) [9.12]. Additional statistical analyses were carried on with the StatView 5.0 software package (SAS Institute Inc, Cary, NC). Anova, unpaired T-Test and when required (specifically, when sample size was inferior to 10 cases in at least 1 group) a non-parametric Mann-Whitney (MW) were adopted for GEP data analyses and, in particular, for comparing *ADFP* expression in different subgroups. The limit of significance for all analyses was defined as *P*<0.05; two-sided tests were used in all calculations.

**Figure 4:**
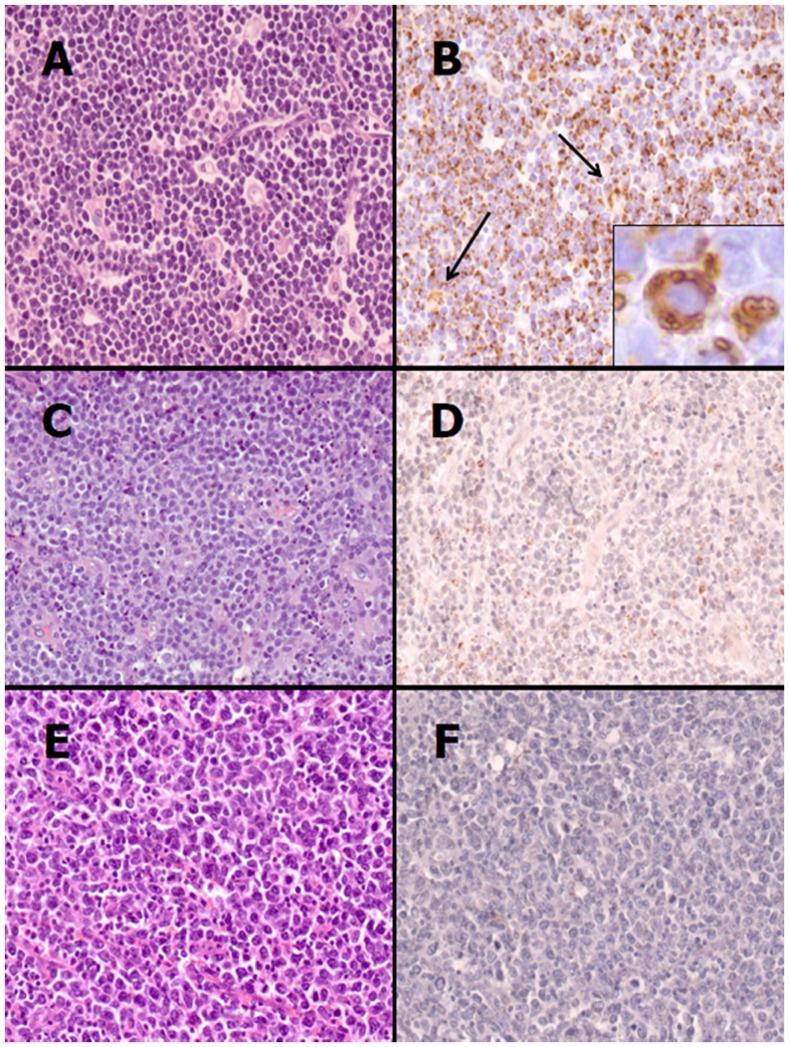
Adipophilin immunostain in BL and not-BL cases. (A) BL is characterized by medium-sized cells with a monotonous cohesive pattern of growth, round nuclei with finely clumped and dispersed chromatin, a high proliferation rate and “starry-sky” appearance [Haematoxilin-eosin (H&E), original magnification (O.M.) ×200). (B) Neoplastic cells show strong positivity to adipophilin with single or multiple droplets in the cytoplasm, sometimes clustering the outer nuclear membrane (inset); the internal positive control is represented by macrophages which show granular positivity in the cytoplasm (arrows) (Adipophilin stain, O.M. ×200; inset, O.M. ×400). (C) An aggressive B-cell lymphoma with diffuse proliferation of medium- to large-sized cells with irregular nuclear contours and relatively large nucleoli corresponding to morphological score 1 according to the Naresh *et al.* scoring system is shown. Few small lymphocytes and starry-sky macrophages are also present (H&E, O.M. ×200). (D) Adipophilin immunostain on the case depicted in figure C with neoplastic cells showing weak positivity characterized by singly scattered fine lipid droplets in the cytoplasm (Adipophilin stain, O.M. ×200). (E) Morphological features of a diffuse large B-cell lymphoma (H&E, O.M. ×200). (F) Adipophilin immunostain on the case depicted in figure E is entirely negative (Adipophilin stain, O.M. ×200).

## Results

### ADFP (PLIN2) Gene is More Expressed in Burkitt Lymphoma than in DIFFUSE Large B-cell Lymphoma

A previous GEP study on BL cases revealed that adipophilin is the only member of the PAT-proteins family expressed in BL [9]. Using the same set of data, we measured the expression of *ADFP* in BL and DLBCLs and found a significant over-expression in the former. Specifically, BL presented significant higher level than both germinal center (GCB)-DLBCL (p = 0.04) and activated B-cell (ABC)-DLBCL (p = 0.03), while no difference was recorded among the DLBCL subtypes (Figure 2). Moreover, supervised analysis revealed that 5 other genes involved in lipid metabolism were differentially expressed (fold change >2; p<0.05) in BL and DLBCL. While *FASN*, *SCD5* and *USF1* were up-regulated in BL, *SCD* and *PPARA* were up-regulated in DLBCLs. (Figure 3; Table 1).

### Adipophilin is Strongly Expressed in Burkitt Lymphoma Cases

To confirm GEP results, adipophilin expression was investigated by immunohistochemistry in BL and not-BL cases, classified according to the recent algorithm proposed by Naresh *et al.* [10]. Distinct patterns of adipophilin expression, highlighting lipid vacuoles, were observed among the two different categories of lymphoma. A strong immunoreactivity, characterized by single or multiple droplets in the cytoplasm and clustering of these to the outer nuclear membrane, was observed in 45 out of 47 BL cases (Figure 4A-B). In these cases, smaller lipid droplets were also present. Weak positivity characterized by dispersed fine lipid droplets in the cytoplasm of a small minority of cells was detected in 3 out of 33 cases of the not-BL category (Figure 4C-D). 30 out of 33 not-BL cases did not show any expression of adipophilin (Figure 4E-F). Macrophages showed a granular staining within the cytoplasm, and this served as an internal positive control. The evaluation of immunohistochemical expression of adipophilin is summarized in table 2. The proportion of cases positive for adipophilin expression was significantly higher (p<0.05) in BL cases than in the not-BL cases.

Interestingly, the three cases of the not-BL category that showed weak, fine positivity were characterized by a diffuse proliferation of medium- to large-sized cells with irregular nuclear contours and relatively large nucleoli. Starry-sky macrophages, mitoses and apoptosis were prominent, and reactive small lymphocytes were scanty. This morphological features corresponded to score 1 (range: 0–3) on the algorithm for aggressive B-cell lymphomas proposed by Naresh *et al.* [10] and may well represent B cell lymphoma, unclassifiable with features intermediate between DLBCL and BL [11].

## Discussion

BL is an aggressive B-cell lymphoma with a worldwide distribution. None of the histological, immunohistochemical or molecular parameters can be singly used for the diagnosis of BL and the WHO classification suggests that a combination of several techniques is necessary [1]. BL is a potentially curable malignancy if correctly diagnosed, even in the resource-poor settings where BL is the most common malignancy in paediatric patients [2]. In these settings, often, performing biopsies is not feasible and the diagnosis of BL is made on FNAC specimens by identifying the cytoplasmic lipid vacuoles within the lymphoid cells. However, FNAC is not always adequate for accurate diagnosis and cytological specimens do not support further investigations [3]. In this study, we have attempted to find a novel marker that can detect lipid vacuoles in histological samples by analysing the lipid metabolism in BL at both gene and protein level.

GEP showed that *FASN*, *SCD5*, *USF1* are up-regulated in BL whereas *SCD* and *PPRA* are down-regulated. In addition, we identified the adipophilin as the only member of PAT-proteins family to be significantly over-expressed in BL [9]. PAT-proteins is a family of lipid droplet-associated proteins which also includes adipophilin, perilipin and TIP47. These proteins are involved in the formation, maintenance, modification and involution of lipid droplets [13]–[14]. While the expression of perilipin is thought to be restricted to adipocytes and certain steroidogenic cells, adipophilin and TIP47 are nearly ubiquitously expressed [15]. In particular, adipophilin is localized to the surface of lipid droplets [4] and is mainly involved in fatty acids transport and in preserving the cellular triacylglycerols content [4], [16]–[17]. Tumour cells require *de novo* synthesis of lipids for membrane assembly and biosynthesis of other macromolecules (such as proteins and nucleic acids) to rapidly dividing [18]. Newly generated fatty acids are promptly incorporated into membrane lipids and triacylglycerol stores to accommodate the dramatic proliferative rates of some types of neoplastic cells [19]. Adipophilin over-expression in BL may thus reflect the up-regulation of lipogenic pathway and might be related to the high proliferation rate of this tumour, the fastest growing tumour in humans.

The results generated by GEP, were validated at protein level by immunohistochemistry using an antibody against adipophilin. BL was characterized by strong immunoreactivity and by the presence of single or multiple droplets in the cytoplasm and clustering of these droplets to the outer nuclear membrane whereas DLBCL showed no expression of adipophilin. A weak labelling may be observed in not-BL cases with features intermediate between DLBCL and BL. Macrophages showed a granular staining in the cytoplasm and this may explain the partial overlap between BL and DLBCL observed at RNA level. These findings suggest adipophilin as a novel marker that can be useful for the diagnosis of BL in histological sections and may be a reliable marker in challenging cases such as DLBCL/BL.

In this paper we gave evidence, for the first time, that lipid metabolism is altered in BL. The extent to which metabolism plays a role in lymphomagenesis should not be underestimated. The change in metabolism cannot be purely attributed to alteration in allosteric and product/substrate regulation of the metabolic enzymes [20]–[24]. Metabolic reprogramming in neoplastic cells involves numerous genes (i.e. *MYC*, *P53*, *H1F1*, *PTEN*) that finally enhances anaerobic glycolysis, lactate production, and biosynthesis of lipids and other macromolecules [19]. The genes that regulate these metabolic pathways may thus serve as targets for specific therapies [23] and, among these, adipophilin may be an interesting candidate for interventional strategies (e.g. target therapy or vaccine therapy). A preliminary study has, in fact, investigated the possible use of adipophilin as a T-cell epitope to induce antigen-specific cytotoxic T-lymphocytes and mediate tumour cell lysis [25].

A better knowledge of lipid metabolism alteration in BL can potentially provide new markers to improve diagnosis and prognosis as well as novel therapeutic approaches for BL treatment [26].

## References

[1] Leoncini L, Raphael M, Stein H, Harris NL, Jaffe ES, et al.. (2008) Lymphoma In: World Health Organization Classification of Tumours Haematopoietic and Lymphoid tissues. IARC Press: Lyon, France, p.262.

[2] Bellan C, Lazzi S, De Falco G, Rogena EA, Leoncini L (2010) Burkitt lymphoma versus diffuse large B-cell lymphoma: a practical approach. Hematological Oncology 28: 53–56. 19844983 10.1002/hon.916

[3] Drut R, Drut RM, Pollono D, Tomarchio S, O Ibáñez, et al (2005) Fine-needle aspiration biopsy in pediatric oncology patients: a review of experience with 829 patients (899 biopsies). J Pediatr Hematol Oncol 27: 370–376. 16012326 10.1097/01.mph.0000173177.40894.8d

[4] Ostler DA, Prieto VG, Reed JA, Deavers MT, Lazae AJ, et al (2010) Adipophilin expression in sebaceous tumors ans other cutaneous lesions with clear cell histology: an immunohistochemical study of 117 cases. Modern Pathology 23: 567–573. 20118912 10.1038/modpathol.2010.1

[5] Straub BK, Herpel E, Singer S, Zimbelmann R, Breuhahn K, et al (2010) Lipid droplet-associated PAT-proteins show frequent and differential expression in neoplastic steatogenesis. Modern Pathology 23: 480–492. 20081801 10.1038/modpathol.2009.191

[6] Tennant DA, Durán RV, Boulahbel H, Gottlieb E (2009) Metabolic transformation in cancer. Carcinogenesis 30: 1269–1280. 19321800 10.1093/carcin/bgp070

[7] Robenek H, Robenek MJ, Troyer D (2005) PAT family proteins pervade lipid droplet cores. J. Lipid Res 46: 1331–1338. 15741656 10.1194/jlr.M400323-JLR200

[8] Bhatt AP, Jacobs SR, Freemerman AJ, Makowski L, Rathmell JC, et al.. (2012) Dysregulation of fatty acid synthesis and glycolysis in non-Hodgkin lymphoma. PNAS May 28 [Epub ahead of print]. 10.1073/pnas.1205995109 PMC3406848 22752304

[9] Piccaluga PP, De Falco G, Kustagi M, Gazzola A, Agostinelli C, et al (2011) Gene expression analysis uncovers similarity and differences among Burkitt lymphoma subtypes. Blood 117: 3596–3608. 21245480 10.1182/blood-2010-08-301556

[10] Naresh KN, Hazem AHI, Lazzi S, Rince P, Onorati M, et al. (2011) Diagnosis of Burkitt Lymphoma using an algorithmic approach-applicable in both resource-poor and resource-rich countries. BJH 2011 Jul 1 [Epub ahead of print]. 10.1111/j.1365-2141.2011.08771.x 21718280

[11] de Jong D (2009) Novel lymphoid neoplasms–the borderland between diffuse large B-cell lymphoma and Burkitt's lymphoma. Haematologica 94: 894–96. 19570750 10.3324/haematol.2009.008128 PMC2704298

[12] Piccaluga PP, Califano A, Klein U, Agostinelli C, Bellosillo B, et al (2008) Gene expression analysis provides a potential rationale for revising the histological grading of follicular lymphomas. Haematologica 93: 1033–1038. 18492688 10.3324/haematol.12754

[13] Brasaemle DL, Barber T, Wolins NE, Serrero G, Blanchette-Mackie EJ, et al (1997) Adipose differentiation-related protein is an ubiquitously expressed lipid storage droplet-associated protein. J Lipid Res 38: 2249–2263. 9392423

[14] Heid HW, Moll R, Schwetlick I, Rackwitz HR, Keenan TW (1998) Adipophilin is a specific marker of lipid accumulation in diverse cell types and diseases. Cell Tissue Res 294: 309–321. 9799447 10.1007/s004410051181

[15] Wolins NE, Rubin B, Brasaemle DL (2001) TIP47 associates with lipid droplets. J Biol Chem 276: 5101–5108. 11084026 10.1074/jbc.M006775200

[16] Muthusamy K, Halbert G, Roberts F (2006) Immunohistochemical staining for adipophilin, perilipin and TIP47. J Clin Pathol 59: 1166–1170. 16556662 10.1136/jcp.2005.033381 PMC1860497

[17] Fujimoto T, Ohsaki Y, Cheng J, Suzuki M, Shinohara Y (2008) Lipid droplets: a classic organelle with new out. Histochem Cell Biol 130: 263–279. 18546013 10.1007/s00418-008-0449-0 PMC2491702

[18] Atshaves BP, Storey SM, McIntosh AL, Petrescu AD, Lyuksyutova OI, et al (2001) Sterol carrier protein-2 expression modulates protein and lipid composition of lipid droplets. J. Biol. Chem. 276: 25324–25335. 10.1074/jbc.M100560200 11333258

[19] DeBerardinis RJ, Lum JJ, Hatzivassiliou G, Thompson CB (2008) The biology of cancer: metabolic reprogramming fuels cell growth and proliferation. Cell Metab (7) 11–20. 10.1016/j.cmet.2007.10.002 18177721

[20] Vander Heiden MG, Cantley LC, Thompson CB (2009) Understanding the Warburg effect: the metabolic requirements of cell proliferation. Science 324: 1029–1033. 19460998 10.1126/science.1160809 PMC2849637

[21] Menendez JA, Lupu R (2007) Fatty acid synthase and the lipogenic phenotype in cancer pathogenesis. Nat Rev Cancer 7: 763–777. 17882277 10.1038/nrc2222

[22] Yamashita T, Honda M, Takatori H, Nishino R, Minato H, et al (2009) Activation of lipogenic pathway correlates with cell proliferation and poor prognosis in hepatocellular carcinoma. J Hepatol 50: 100–110. 19008011 10.1016/j.jhep.2008.07.036

[23] Kuhajda FP (2000) Fatty-acid synthase and human cancer: new perspectives on its role in tumour biology. Nutrition 16: 202–208. 10705076 10.1016/s0899-9007(99)00266-x

[24] Swinnen JV, Brusselmans K, Verhoeven G (2006) Increased lipogenesis in cancer cells: new players, novel targets. Curr Opin Clin Nutr Metab Care 9: 358–365. 16778563 10.1097/01.mco.0000232894.28674.30

[25] Schmidt SM, Schag K, Mü ller MR, Weinschenk T, Appel S, et al.. (2004) Induction of Adipophilin-Specific Cytotoxic T Lymphocytes Using a Novel HLA-A2-Binding Peptide That Mediates Tumor Cell Lysis. Cancer research 64, 1164–1170. 10.1158/0008-5472.can-03-2538 14871853

[26] Onnis A, De Falco G, Antonicelli G, Onorati M, Bellan C, et al (2010) Alteration of microRNAs regulated by c-Myc in Burkitt lymphoma. Plos One 5(9): 1–9. 10.1371/journal.pone.0012960 PMC2945769 20930934

